# Clinical Manufacturing of Regulatory T Cell Products For Adoptive Cell Therapy and Strategies to Improve Therapeutic Efficacy

**DOI:** 10.1080/15476278.2022.2164159

**Published:** 2023-01-22

**Authors:** Kassandra J. Baron, Hēth R. Turnquist

**Affiliations:** a Departments of Surgery, University of Pittsburgh School of Medicine, Pittsburgh, Pennsylvania, USA; b Thomas E. Starzl Transplantation Institute, University of Pittsburgh School of Medicine, Pittsburgh, Pennsylvania, USA; c Department of Infectious Disease and Microbiology, University of Pittsburgh School of Public Health, Pittsburgh, Pennsylvania, USA; d Department of Immunology, University of Pittsburgh School of Medicine, Pittsburgh, Pennsylvania, USA; e McGowan Institute for Regenerative Medicine, University of Pittsburgh, Pittsburgh, Pennsylvania, USA

**Keywords:** Regulatory T cells, GMP manufacturing, *ex vivo* expansion, solid organ transplant, immunotherapy

## Abstract

Based on successes in preclinical animal transplant models, adoptive cell therapy (ACT) with regulatory T cells (Tregs) is a promising modality to induce allograft tolerance or reduce the use of immunosuppressive drugs to prevent rejection. Extensive work has been done in optimizing the best approach to manufacture Treg cell products for testing in transplant recipients. Collectively, clinical evaluations have demonstrated that large numbers of Tregs can be expanded *ex vivo* and infused safely. However, these trials have failed to induce robust drug-free tolerance and/or significantly reduce the level of immunosuppression needed to prevent solid organ transplant (SOTx) rejection. Improving Treg therapy effectiveness may require increasing Treg persistence or orchestrating Treg migration to secondary lymphatic tissues or places of inflammation. In this review, we describe current clinical Treg manufacturing methods used for clinical trials. We also highlight current strategies being implemented to improve delivered Treg ACT persistence and migration in preclinical studies.

## Introduction

Regulatory T cells (Tregs) are a specialized subpopulation of CD4^+^ T cells that are crucial in maintaining immune homeostasis and preventing autoimmunity.^1^ Tregs in humans are characterized by their prolific expression of the high-affinity interleukin IL-2 receptor α-chain (CD25), transcription factor forkhead box P3 (Foxp3), and low expression of IL-7 receptor α-chain (CD127).^2^ The importance of Treg number and/or function in immune tolerance is illustrated by immunodysregulation polyendocrinopathy enteropathy X-lined (IPEX) syndrome which is caused by a mutation in the FoxP3 gene and can be cured by restoring normal Treg populations via hematopoietic cell transplant (HCT).^3,4^

Tregs function to suppress immune responses toward self and non-self by limiting the activation and proliferation of other T cells through multiple mechanisms. These mechanisms include the production of anti-inflammatory cytokines such as TGF-β (transforming growth factor beta) and IL-10 and the expression of membrane-bound molecules such as CTLA-4 (cytotoxic T cell-associated antigen), LAG-3 (lymphocyte activation gene 3), TIGIT (T cell immune receptor with Ig and ITIM domains), and CD39 (ectonucleoside triphosphate diphosphohydrolase 1).^1,5^ Tregs can also modulate antigen-presenting cells (APCs) through contact-dependent mechanisms that alter APCs capacity for co-stimulation and antigen presentation.^5^ Additionally, high expression of CD25 enables them to sequester local IL-2 which limits effector T cell expansion and function by depriving them of IL-2.^6,7^

Interest in harnessing the suppressive capacity of Tregs for immunotherapy originates from early studies which demonstrated the importance of CD4^+^CD25^+^ Tregs in inhibiting the development of autoimmune disease.^8^ It was then demonstrated that naturally occurring CD4^+^CD25^+^ Treg populations could be expanded *ex vivo* to treat autoimmune diseases in mice by suppressing and modulating effector T cells.^9^ Thereafter, using adoptively transferred CD4^+^CD25^+^ Tregs in a series of preclinical sentinel model studies in solid organ transplantation (SOTx) and graft-*versus*-host disease (GvHD) after allogeneic hematopoietic cell transplantation (AlloHCT) further supported Treg therapeutic potential to induce immunological tolerance to non-self-antigens to prevent allograft rejection and GvHD. GvHD is a life-threatening complication of AlloHCT, which is used to treat hematological and non-hematological diseases.^10^ GvHD is an alloreactive donor T cell-mediated response driven immunologically by differences between donor and recipient. In seminal studies completed by Taylor *et al*., they demonstrated that depletion of murine CD4^+^CD25^+^ cells from the BM donor increased GvHD, which was then reduced upon infusion of fresh donor CD4^+^CD25^+^ cells.^11^ Since then, utilizing Treg to prevent/delay GvHD has been routinely demonstrated in preclinical murine models.^12–14^

The therapeutic potential of Tregs to support allograft survival after SOTx has also been demonstrated using preclinical models of skin and heart Tx. For example, heart allograft survival was prolonged following transfer of *ex vivo* expanded CD4^+^CD25^+^ Tregs in wild-type mice.^15^ Additionally, studies using humanized murine models demonstrated prolonged allograft survival in pancreatic islet and skin Tx following adoptive transfer of huTreg.^16–18^ What would further translate to huTregs was the finding that murine Treg function was more potent than CD4^+^CD25^+^ Treg when sorted based on low expression of CD127.^19^ Due to their immunological similarity to humans, the use of non-human primate models (NHP) has also been important in evaluating adoptive cell therapy (ACT) with Treg in SOTx. *Ex vivo* expanded Treg in NHP models are well characterized and adoptive transfer has been reported to suppress renal allograft rejection and prolong survival.^20–22^

Recently, clinical studies have been completed or underway assessing Treg to improve outcomes after SOTx or with AlloHCT to prevent GVHD.^5, 23–25^ These clinical studies have also shown that large numbers of CD4^+^CD25^+^ Treg can be expanded in the presence of high doses of IL-2 *ex vivo* and infused safely.^5, 26–34^ The number of clinical trials utilizing Treg cell therapy in transplantation and other fields are expected to increase. In this review, we describe current clinical GMP methods used to isolate and expand Tregs *ex vivo* for clinical trials in solid organ Tx and treatment/prevention of GvHD. We then discuss *in vivo* and *ex vivo* strategies being used to improve adoptive Treg cell therapy persistence and migration to increase the efficacy of Treg therapy in preclinical studies. While numerous preclinical publications exist describing manufacturing methods of Treg cell products, few of these publications provide a correlation with completed clinical studies in SOTx. Therefore, to improve and enhance Treg cell therapy in the clinic, it is important to highlight manufacturing protocols specific to completed and recruiting clinical trials when possible. In this review, we aim to briefly introduce general concepts around Treg and then highlight clinical manufacturing methods used to generate Treg cell products used in SOTx referencing published clinical trial protocols, manuscripts, and preclinical protocol development. It is also noteworthy that even in preclinical animal transplant models Treg ACT has only provided prolonged SOTx survival. Thus, even at the preclinical level, Treg ACT is far from optimized. All clinical trials to date unfortunately have not reduced the levels of immunosuppressive drugs needed to prevent SOTx rejection.^5,21,28,33^ Thus, we also provide examples of ways investigators are working to improve Treg ACT and move the needle toward the field’s goal of maintenance of normal graft function without the use of immunosuppression, also known as drug-free tolerance or operational tolerance (OT).

### Adoptive Treg therapy

Treg ACT has developed due to the need for alternative therapeutic agents to limit the need for immunosuppression (IS) after SOTx, and ideally support the routine induction and maintenance of immune transplant tolerance in recipients of allogeneic materials. SOTx remains the only effective lasting treatment for end-stage organ diseases. Strategies to increase short-term outcomes after SOTx have relied on better patient donor selection and improved immunosuppressive regimens.^24^ For example, calcineurin inhibitors, antiproliferative agents, and mTOR (mammalian target of rapamycin) inhibitors can control the immune response early post-transplant, but have detrimental side effects such as cardiovascular diseases, kidney failure, and susceptibility to opportunistic infections.^24^ Efforts to improve long-term outcomes and 10-year survival remain unsuccessful due to the failure of multi-drug immunosuppression to address chronic rejection despite their toxic side effects.^21,35^ Other therapeutic strategies such as co-stimulation-blockade (CoSB) or monoclonal antibody (Ab) therapy to target cytokines and other co-stimulatory molecules have only had limited success at reducing the use of immunosuppressive drugs.^21^ Therefore, utilizing Tregs or other regulatory immune cells as a potential immune cell therapy may be a promising alternative to reduce the use of immunosuppressive drugs and even induce allograft tolerance based on data obtained from various preclinical murine and NHP studies.

Despite new prophylaxis strategies, GVHD is a common side effect of AlloHCT where alloreactive T cells destroy host tissues. Current treatments for GVHD involve nonspecific multi-drug immunosuppression, particularly corticosteroids, that often leads to morbidity and mortality due to cancer relapse or secondary infection.^10^ Thus, alternative therapies such as Tregs are being investigated to prevent the development of GVHD after AlloHCT. If prophylaxes therapies can successfully prevent GVHD, this could also expand the use of AlloHCT beyond cancer treatment and for routine use as a means to induce transplant tolerance after SOTx.^36–38^

### Treg isolation

The donor source of Tregs is important when designing and describing Treg cell therapy products. Tregs are typically classified as thymic-derived Tregs (tTreg) and peripherally-derived Tregs (pTreg).^39^ In contrast to tTreg cells that originate from the thymus, pTreg develop from conventional CD4^+^ T cells in the periphery after antigen encounter along with TGF-β, especially in the gut.^23^ Similarly, investigators have “induced” Foxp3^+^ Treg (iTreg) *in vitro* from CD4^+^ Foxp3- populations by expanding CD4^+^CD25^−^ cells in the presence of IL-2, rapamycin, and TGF-β.^40^ To date, the majority of the Tregs used in clinical trials for SOTx, however, were isolated from autologous peripheral blood mononuclear cells (PBMC) or Umbilical cord blood (UCB). Unfortunately, there are no consistently reliable specific markers to distinguish between tTreg and pTreg in mice and humans, thus making specific origination of Treg isolated from these sources imprecise. There is evidence to support the use of allogenic Tregs in humans; however, that is beyond the scope of this review.^26,27,30^ Therefore, this review will focus discussions on the detailed manufacturing of autologous Tregs from human subjects.

Human Tregs are relatively rare in the PBMCs and comprise only 2–10% of peripheral CD4^+^ T cells in healthy adults. Thus, Treg isolation and *ex vivo* expansion is required to reach the numbers of Treg predicted in animal studies to be therapeutic after adoptive transfer.^21,41,42^ As introduced above, the Tregs for clinical trials have been most commonly isolated from PBMC or UCB.^24,26,27, 43–45^ A recent novel strategy used pediatric thymuses which are routinely discarded during pediatric heart transplantation as a Treg source.^46,47^ Tregs develop in the thymus and thus the thymus represents an attractive alternative source to isolate Tregs for cellular therapies. In a study involving 11 different donors, roughly 12% of the CD4^+^ cells were CD3^+^ CD4^+^ CD8^−^ CD25^+^ CD127^−^ with 85% of these cells expressing the FoxP3.^46^ This approach is an exciting alternative to isolate large numbers of Tregs; however, it is so far limited to pediatric heart transplant recipients or when third-party Tregs are utilized. The clinical feasibility to isolate and expand functional tTregs is currently being explored in an ongoing clinical trial (THYTECH; NCT0492449) where tTregs isolated from the thymus are being tested for their ability to prevent rejection after pediatric heart transplantation.^46^

Treg isolation from leukapheresis has also been evaluated as a potential alternative source and offers increased starting cell density to improve Treg yields for subsequent *ex vivo* expansion.^48^ Although not yet tested in clinical trials, the use of Granulocyte Colony-Stimulating Factor (G-CSF)-mobilized peripheral blood stem cells (G-PBSC) may be another potential source that could be used to isolate Tregs for patients receiving HCT.^49,50^ In this case, Treg isolation and expansion would be performed after CD34 selection utilizing the typically discarded CD34 negative fraction. Application of G-CSF significantly increased Treg yield while preserving suppressive function and phenotype.^50^ To date, only PBMC and UBC have been clinically tested in SOTx and AlloHCT.

There has been rapid progress in the development of Good Manufacturing Practice (GMP) grade protocols to isolate and expand large numbers, and recent reviews have detailed the strategies used to isolate Tregs from various blood products for use in clinical trials.^24,44,51,52^ Of these, common isolation techniques include fluorescence-activated cell sorting (FACS), direct or untouched magnetic cell separation, or a combination of both. In the case of magnetic cell separation, these systems utilize magnetic microbeads to bind cells of interest (direct enrichment, or “positive” selection) or to selectively deplete unwanted cells (“negative” selection). Additionally, regardless of the methodology or source, high surface expression of CD25 remains the primary marker used to distinguish human Tregs from non-target cells along with low expression of CD127.^53^ Unfortunately, there is no singular surface marker for Tregs that distinguish them from other immune cells, and Foxp3 is an intracellular transcription factor that cannot be utilized for the isolation of live cells. Therefore, multiple surface parameters are needed to isolate Treg from blood products. Many groups have utilized cell sorting based on parameters of CD3^+^CD4^+^CD25^+^CD127^low^.^28,31, 54–56^ While this process yields highly pure Tregs, it is less efficient as run times can take many hours depending on the purity and cell concentration of the starting product. Additionally, there can be considerable cell loss with traditional sorting via FACS or positive and negative bead selection. Such loss impacts the overall product yield and increases the need for robust *ex vivo* expansion. These shortcomings have driven advancements that have been made in streamlining GMP-compliant protocols to generate clinical grade Tregs.

One of the most notable advancements in this field has been with Miltenyi’s closed system magnetic cell separators CliniMACS Prodigy and CliniMACS Plus.^57^ These systems utilize magnetic microbeads to enrich for Tregs or to selectively deplete non-Treg (CD19^+^, CD8^+^, or CD127^+^ cells). Like flow cytometric cell sorting, this platform allows for multiparameter selection, and numerous protocols specifically for Tregs have been developed and established. Currently, the approaches most cited are CD25 enrichment with or without prior selective depletion of CD8^+^ and/or CD19^+^ cells.^26,27,29,30,48, 58–63^

Another notable strategy that is becoming more common for Treg isolation allowing for a high purity Treg product is CD25 enrichment utilizing Miltenyi’s CliniMACS systems as described above in combination with a purification based on CD4^+^CD25^+^ CD127^low^ selection using the closed cartridge, low cost MACSQuant® Tyto® (Miltenyi Biotec: Bergisch Gladbach, Germany). This strategy works by using a clinical GMP grade CD25 reagent (like the CD25 Enrichment microbeads) that is also biotinylated which allows the user to fluorescently label CD25 positive cells with phycoerythrin (PE) and perform purification using the MACSQuant Tyto sorter following the CD25 enrichment step. In addition, other fluorescent markers in addition to CD25, such as CD4, CD127, or CD45RA, can be used for subsequent sorting if desired. This approach significantly reduces the processing time while also preserving the yield and purity of the product. If optimized, this type of processing approach could limit product variability and increase reliability. Investigators have demonstrated the feasibility of using the CliniMACS for CD4^+^CD25^+^ Treg enrichment; however, in this case, the purity was suboptimal.^64,65^ In theory, subsequent sorting for CD4^+^CD25^+^CD127^low^ could significantly improve the purity and allow for robust *ex vivo* expansions of pure Treg populations.

### Treg expansion

Based on the opinion, large doses of Tregs will be the most effective immunotherapy. CliniMACS-based and other Treg isolation protocols have been paired with *ex vivo* Treg expansion. There have been numerous different reagents and approaches used for generating clinical Treg products for the use in kidney transplantation (Table 1) and liver and heart transplantation (Table 2). Tables 1 and 2 summarize completed and recruiting Phase I/II clinical trials in SOTx and the associated Treg manufacturing protocols. Using the isolation methods mentioned above, many groups have demonstrated that high numbers of Tregs can be isolated and subsequently expanded. Of note, it is evident in Tables 1 and 2 that there is a considerable lack of data on the purity and Treg number resulting from most isolation protocols. This is unfortunate as it is important to understand Treg viability, phenotype, and purity through isolation steps as it correlates to end product stability, function, as well as relevant clinical observations.

**Table 1.** Published manufacturing protocols for regulatory T cells assessed in kidney transplantation phase I/II clinical trials.

Kidney transplantation										
Trial ID/Name	Phase/Study status	Ref.	Treg product (name)	Target dose	Isolation method	Expansion/Culture conditions	Activation reagent	Expansion length	Purity or release criteria	Outcome
NCT02129881 (ONE)*	I/II (Completed)	59, 98, 114, 115	polyTreg	1–6 x 10^6^/kg	CliniMACS CD8^+^ depletion & CD25^+^ Enrichment	TexMACS media + 5% HS + 500 IU/mL IL-2 + 100 nM Rapa	MACS GMP ExpAct Treg kit (Miltenyi)	36 days w/ restim d12 and 24	74.7–88.2% CD4^+^CD25^+^FoxP3^+^	No adverse effects in 12 patients.
NCT02088931 (TASK)*	I (Completed)	28	polyTreg	3.2x10^8^ total cells	FACS for CD4^+^CD25^high^CD127^low^	X-Vivo 15 + 10% HS + 300 IU/mL IL-2 (day 2)	anti-CD3/28 beads (Invitrogen)	14 days w/ restim d9	93–96.7% FoxP3^+^	Infused Tregs were stable and persistent. No adverse events or infusion reactions. Tregs peaked in circulation in the first week and were detectable during first month dropping off at 3 months post infusion.
NCT02145325 (TRACT)*	I (Completed)	29	polyTreg	0.5–5.0 × 10^9^ total cells	CliniMACS CD8^+^ depletion & CD25^+^ Enrichment	TexMACS + 5% HS + 1 ug/mL TGF-B + 1000 IU/mL (on day 0) + 100 ng/mL Rapa (days 0–9)	MACS GMP ExpAct Treg kit (Miltenyi)	21 days w/ restim d7	>80% FoxP3^+^	All doses of Tregs were safely infused with no infusion related side effects. No infections or rejection events up to two years post Tx.
NCT02371434 (ONEnTreg13) *	I/II (Completed)	98, 114	polyTreg	0.5–3 x 10^6^/kg	CliniMACS CD8^+^ depletion & CD25^+^ Enrichment	X-Vivo 15 + 10% HS + 500 U/mL IL-2 + 100 nM Rapa & several re-stims	MACS GMP ExpAct Treg kit (Miltenyi)	23 days	>90% CD4+ CD25+ FoxP3+ of total cells	No dose limiting toxicity observed. Treg cell therapy is achievable and safe in living-donor kidney transplant recipients.
NCT02244801 (ONE) (DART)*	I/II (Completed)	89, 98	darTreg	3.0 or 9.0 × 10^8^ total cells	FACS for CD4^+^CD25^high^CD127^low^	OpTimizer Medium + GlutaMAX + Penicillin/Streptomycin + 2% HS or X-Vivo15 medium + 10% HS with 300 IU/mL IL-2	sBcs (4:1 sBC:T cell) or GMP anti-CD3/28 beads (1:1 bead:cell)	Restim d9 or d11	*Not specified*	Part of the ONE study. Treg therapy is safe in living-donor kidney transplant recipients.
NCT02091232 (ONE)*	I/II (Completed)	98	Belatacept-conditioned darTreg	3.0 or 9.0 x 10^8^	Magnetic bead–selected Tregs generated by *ex vivo* belatacept-mediated CSB	PBMCs were co-cultured for 72hrs with an equal number of irradiated kidney donor PBMNCs + Belatacept and restim without CSB	*Not specified*	*Not specified*	*Not specified*	Part of the ONE study. Treg therapy is safe in living-donor kidney transplant recipients.
NCT02711826 (TASK)*	I (Recruiting)	28, 117	polyTreg or darTreg	3.0–5.0 × 10^6^ total cells	*Not specified*	*Not specified*	*Not specified*	*Not specified*	*Not specified*	*n.a.*
ISRCTN11038572 (TWO)*	IIB (Recruiting)	24, 59	polyTreg	5–10 x 10^6^/kg	CliniMACS CD8^+^ depletion & CD25^+^ Enrichment	*Not specified*	anti-CD3/28 GMP-grade expansion beads	*Not specified*	*Not specified*	*n.a.*
NCT03284242*	I/II (Recruiting)	118	polyTreg	Unknown	CliniMACS CD4^+^CD25^high^ Enrichment	TexMACS + 2% HS + 500 IU/mL IL-2 + 100 nM Rapa or Evermolis	MACS GMP ExpAct Treg kit (2:1 bead:cell)	*Not specified*	*Not specified*	*n.a.*

*Details of expansion protocol and data obtained from referenced publication describing preclinical or clinical protocol development. Ref. = References; Treg = regulatory T cell; Foxp3 = forkhead box protein 3; CSB = co-stimulatory blockade; sBc = stimulated B cell; Tx = transplant; dar-Treg = donor alloantigen reactive; polyTreg = polyclonal regulatory T cells; w/ = with; restim = restimulation; n.a. = not applicable; HS = suman serum; KTx = Kidney Transplantation; LTx = Liver Transplantation; HTx = Heat Transplantation.

**Table 2.** Published manufacturing protocols for regulatory T cells assessed in liver and heart transplantation phase I/II clinical trials.

Liver Transplantation										
Trial ID/Name	Phase/Study status	Ref.	Treg product (name)	Target dose	Isolation method	Expansion/Culture conditions	Activation reagent	Expansion length	Purity or release criteria	Outcome
NCT02166177 (ThRIL)*	I (Completed)	*33, 45*	polyTreg	0.5–6.5 × 10^6^ cells/kg	CliniMACS CD4^+^CD25^high^ Enrichment	TexMACS + 5% HS + 500 IU/mL IL-2 (day 4) + 100 nM Rapa	MACS GMP ExpAct Treg kit (Miltenyi)	36 days w/ restim every 10–12 days	Mean 89.2% ±2.61% CD4+ CD25+ FoxP3+	Treg administration was safe and well tolerated. Therapy increased the pool of circulating Tregs and reduced anti-donor T cell responses.
UMIN-000015789*	I (Completed)	85	donor-specific iTreg-enriched	0.34–6.37 × 10^6^ cells/kg	Recipient lymphocytes separated by Ficoll-Paque	Lymphocytes co-cultured with irradiated donor PBMCs + 10ug/mL anti-CD80/86 antibodies for 2 weeks in AlyS505N-0 B10 medium and recipient HS + CSB	*Not specified*	14 days	2.6–16.9% of cell population	Treg infusion was safe and immunosuppression withdrawal achieved in 7/10 patients with no significant adverse events.
*NCT02474199 (ARTEMIS)*	I/II (Completed with results)	*25, 94, 116, 117*	darTreg	3.0–5.0 × 10^8^ cells	FACS for CD4^+^CD25^high^CD127^low^	Medium + deuterated glucose in co-cultures with donor sBcs	anti-CD3/28 GMP beads for secondary stimulation	16 days	*>95% CD4, >60% FOXP3, <5% CD8, <1% CD19 by flow cytometry; >85% viable*	Treg infusion was not associated with adverse events. Insufficient number of recipients were treated for assessing efficacy. Four of nine Treg products failed to meet minimal infusible dose.
*NCT03577431 (LITTMUS-MGH)*	I/II (Recruiting)	*25, 33*	*Alloantigen reactive Treg (arTreg-CSB)*	2.5–500 x 10^6^	CD4^+^CD25^high^ Treg	*Not specified*	*Not specified*	*Not specified*	*Not specified*	*n.a.*
NCT03654040 (LITTMUS-UCSF)	I/II (Recruiting)	*25, 116, 117*	darTreg	1.0–5.0 × 10^7^ total cells	FACS for CD4^+^CD25^high^CD127^low^	*Not specified*	*Not specified*	*Not specified*	*Not specified*	*n.a.*
NCT02188719 (deLTa)*	I (Terminated)	*25, 89, 116, 117*	darTreg	25–960 x 10^6^ total cells	FACS for CD4^+^CD25^high^CD127^low^	300 IU/mL IL-2	sBcs (4:1 sBcs:Treg) and anti-CD3/28 beads (1:1 bead:cell)	14 days w/ restim on d9 or d11	≥95% CD4+, ≥60% FOXP3+, ≤5% CD8+, ≤1% CD19+	Enrollment was terminated due to high number of ineligible subjects, slow enrollment, and manufacturing difficulties within the constraints of the funding period.
**Heart transplantation**										
NCT04924491 (THYTECH)*	I/II (Recruiting)	*46*	polyTreg	1.0–2.0 × 10^7^ thyTreg/kg	CliniMACS CD25 Enrichment	TexMACS GMP medium + 600 IU/mL IL-2	T cell TransAct (Miltenyi)	7–10 days	70.10% – 98.41% of xfCD25+ FOXP3+ cells	*n.a.*

*Details of expansion protocol and data obtained from referenced publication describing preclinical or clinical protocol development. Ref. = References; Treg = regulatory T cell; Foxp3 = forkhead box protein 3; CSB = co-stimulatory blockade; sBc = stimulated B cell; Tx = transplant; dar-Treg = donor alloantigen reactive; polyTreg = polyclonal regulatory T cells; w/ = with; restim = restimulation; n.a. = not applicable; HS = suman serum; KTx = Kidney Transplantation; LTx = Liver Transplantation; HTx = Heat Transplantation.

Human Tregs can be expanded in several ways all of which are dependent on IL-2 for proliferation and maintenance of the Treg phenotype (CD4^+^CD25^+^CD127^low^FoxP3^+^). There is variation among protocols, however. IL-2 is typically added at day zero at 1000 U/mL and replenished as needed.^44,52^ There is no standard IL-2 concentration; however, a recent review has provided a detailed summary of reported concentrations (300–1000 U/mL) used for expansion in clinical trials.^44^ The use of mTOR inhibitor rapamycin, an immunosuppressive drug used to prevent graft rejection, can also be used in clinical grade Treg expansion protocols.^29,30,45,55,57,59,66^ Rapamycin selectively supports the *ex vivo* expansion of CD4^+^CD25^+^FoxP3^+^ Tregs while limiting the proliferation of contaminating non-Treg. Likewise, rapamycin promotes the stability and functional capacity of expanded Treg to suppress the proliferation of both autologous and allogenic CD4^+^ and CD8^+^ T cells *in vitro*.^67^ This was also confirmed in preclinical rodent studies demonstrating that Tregs could be expanded *ex vivo* in the presence of rapamycin, and rapamycin delivery with Treg ACT can support tolerance to SOTx in rodents.^68–71^ Therefore, in cases with less than ideal Treg purity following isolation, it may be beneficial to expand Tregs in the presence of rapamycin to block proliferation of unwanted CD4^+^CD25^−^ cells.

### Stimulating/activating strategies

T cell receptor (TCR) engagement is required for Treg cell differentiation and the induction of Foxp3.^72,73^ Recent studies in mice allowing the deletion of the Treg TCR have also revealed critical functions for TCR signaling in Treg lineage maintenance and their suppressive function.^74–77^ In addition to TCR signaling, CD28 co-stimulation is necessary for Treg activation, proliferation, and function. Early experiments using mice deficient for CD28 or CD80/86 found reduced Treg populations, decreased CD25 expression, and suggested an important role for co-stimulation in Treg survival, FoxP3 expression, and suppressor function.^78,79^ The lack of CD28 or its ligands, CD80 and CD86, decreased Treg and exacerbates diabetes in nonobese diabetic (NOD) mice.^78^ Additional observations in rodents and humans also demonstrate the importance of CD28 signaling for Treg proliferation.^80–83^ As such, most clinical trials have expanded nonspecific polyclonal Tregs (polyTregs) using CD3/CD28 expander beads which provide both TCR and CD28 co-stimulation. There are several published GMP compatible methods used to *ex vivo* expand Tregs for cell therapy which have been previously described.^5,24,44,52^ The optimal way to stimulate Tregs remains unclear; however, there are two main approaches to *ex vivo* expand polyTregs for clinical trials. The most common approach to expand human polyTregs is to use polyclonal stimulation with anti-CD3 and anti-CD28 coated microbeads.^26, 28–31, 45,54,56,84,85^ GMP grade anti-CD3 and anti-CD28-coated nanoparticles are available for clinical use from *Miltenyi Biotec* (ExpAct™ Treg kit) and *Invitrogen* (CTS Dynabeads™ CD3/CD28). These beads are typically added at the start of culture and are expanded over a period of 14–36 days. It is unclear if multiple restimulations are required. Of those reported, fold expansions have been variable and can range from 100 to 2000 with IL-2 alone.^26,31,54,63^ Tregs can also be activated using artificial antigen-presenting cells (aAPCs). These cells have been developed to replace natural antigen-presenting cells (APCs) which mediate T cell effector function. Using lentiviral vector technology, aAPCs can be generated to more closely replicate the features of DCs that deliver CD28 co-stimulation.^86^ For example, aAPCs expressing CD64 and CD86 were used to expand human UCB-derived Tregs.^27^ These cells are advantageous because they do not require removal post expansion as cells are lethally irritated prior to use. K562 cells expressing CD86 and CD64 that are loaded with soluble anti-CD3 have been used in clinical trials.^27,40^ It has been reported that these aAPCs are highly effective at expanding Treg.^27,60^ For example, Brunstein et al. were able to achieve a 10,000-fold expansion of Tregs from UBC in 2 weeks using the aAPCs that expressed CD64 and CD86. In the presence of rapamycin, 3000-fold expansion was achieved.^87^

In addition to manufacturing polyclonal Tregs through the process discussed above, generating donor-antigen-specific/reactive Tregs (darTreg) has been gaining traction in recent attempts to induce tolerance after SOTx. In comparison to polyTregs, darTregs are expanded in the presence of donor cells and exhibit donor specificity after alloantigen exposure by selectively targeting alloreactive effector T cells that are detrimental to the allograft.^88^ Generally, darTreg are generated by isolating recipient Tregs and culturing them with donor alloantigen-expressing APCs from the transplant donor, such as monocyte-derived DC and B cells.^89,90^ The global Treg pool contains a small fraction (10–20%) of darTregs, and generating them in large quantities has been a barrier for successful implementation.^5,16,21, 91–94^ Despite manufacturing challenges due to low frequency of darTregs, experimental models of transplantation have demonstrated that donor antigen-specific Tregs (darTregs) are more potent than polyTregs in promoting allograft survival.^16,71,88,95^ Therefore, due to increased specificity for alloreactive effector T cells and increased potency, it is likely that fewer darTreg are required to induce tolerance compared to polyTregs.

The first report of successful drug-free tolerance with a donor antigen specific cell therapy product in patients undergoing organ transplantation was published by Todo et al. In this study, investigators isolated recipient and donor lymphocytes and co-cultured recipient lymphocytes with irradiated donor lymphocytes with monoclonal antibodies to CD80/86 without IL-2 or rapamycin.^85^ Patients who underwent living donor liver Tx (LDLT) were lymphodepleted 8–10 days post liver Tx and received a single infusion of the darTreg-enriched product. Of the 10 patients infused, 7 met the trial-defined endpoint of tolerance and were successfully weaned off immunosuppression within the study period.^85^ Overall, infusions of 100–300 million cells were well tolerated without significant adverse event and the mean number of Tregs (CD4^+^CD25^+^FoxP3^+^) infused were 24.8% of the CD4^+^cells, which is a low Treg concentration compared to other clinical studies (Tables 1 and 2).^85^ The investigators measured suppressive function of the expanded cells *in vitro* using the mixed lymphocyte reaction (MLR) and inhibited recipient T cell proliferation by donor antigen stimulation. Yet, it is unclear what cell populations in the darTreg-enriched product were indeed responsible for tolerance induction *in vivo* and suppressive function *in vitro*. Nonetheless, these were impressive and exciting studies. As the Tregs made up a very small fraction of the total product, this would suggest that lower numbers of darTreg are needed to induce tolerance or at the least weaning of immunosuppressive regimens. Additionally, it suggests that possibly other regulatory cells in addition to darTregs may be beneficial, contributing to tolerance induction or reduction of immunosuppression. Todo et al.’s study has facilitated the development of protocols to expand antigen-specific or darTregs from humans.^9,89,93,96,97^ Several clinical trials are testing darTregs in SOTx (Tables 1 and 2). For example, the ONE Study was a massive multicenter study that included two polyTreg products and two darTreg products.^98^

Recently, results of the Artemis Phase I/II clinical trial (NCT02474199) have been published. The objective of this study was to determine safety and efficacy of a single dose of darTreg to facilitate reduction of immunosuppression in patients 2–7 years post liver Tx. Nine participants initiated immunosuppression reduction and were eligible for a single darTreg infusion; however, only 5 of the 9 products manufactured met release criteria.^94^ The products that were not infused failed release criteria due to insufficient dose highlighting the challenge to manufacture enough darTregs for clinical trials. Investigators aimed to infuse a total of 100–500 million darTreg. Two patients received >300 million and the other three received 100–200 million darTregs. In comparison to the study by Todo et al., their product manufactured from starting population Tregs was more pure meeting release criteria of >95% CD4^+^, >60% FoxP3^+^, and <5% CD4^−^CD8^+^.^94^ Of the 5 participants who received darTregs, all infusions were well tolerated with no reported significant adverse events. Two participants met the primary endpoint of 75% reduction of calcineurin inhibition or discontinuation of a second drug, but none of the participants attempted complete immunosuppression withdrawal.^94^ Efficacy could not be assessed due to too few treated participants. However, the investigators’ mechanistic studies offer insights to darTreg function in this patient population and current challenges to overcome. Their studies suggest darTreg are dysfunctional in patients 2–7 years after liver Tx. Mechanistic and phenotypic studies suggest an upregulation in activation, exhaustion, senescence, or progressive deletion after liver transplantation.^94^ To investigate Treg donor reactivity after liver TX, the authors performed *in vitro* studies using blood samples from the AWISH study (AWISH; NTC00135694). Tregs were assessed longitudinally in 16 patients at pre-Tx, 6 months post-Tx, and 2 years post-Tx to assess darTreg and conventional T cell reactivity to donor antigen. Overall, authors showed reduced proliferation and selective reduction of donor reactivity in all T cells after liver Tx starting 6 months post liver Tx persisting until 2 years after transplant.^94^ The differences in epigenetic regulation, transcription, and donor reactivity of darTregs in liver Tx highlight important insights to the difficulties of generating successful Treg products and underpin challenges faced when manufacturing enough darTregs. Future longitudinal studies should investigate both darTreg and polyTreg transcriptional changes and donor reactivity pre- and post-Tx when possible, which may offer solutions to improve clinical manufacturing of Treg products that are functionally potent and persist after infusion.

### Alternative strategies to improve efficacy and Treg *in vivo* persistence

Multiple clinical trials have demonstrated the safety and tolerability of adoptive Treg cell therapy after AlloHCT or SOTx.^5,23,24,31,44^ However, clinical trials attempting to limit recipient anti-AlloAg responses have yet failed to translate to reduced use of immunosuppressants after SOTx.^5,28,32,33^ One factor potentially leading to the limited efficacy of Treg ACT may be due to their reduced persistence *in vivo*. Results from clinical trials demonstrate that Tregs expanded in culture with high doses of IL-2 decrease rapidly *in vivo* and methods of tracking infused Tregs are limited.^54,85,94,99^ Thus, alternative methods such as administering low-dose IL-2 (ld-IL-2) to increase their persistence *in vivo* are being investigated. As discussed in earlier sections, Tregs require the growth factor IL-2 for proliferation, survival, and functional activity.^43^ Additionally, Treg ACT requires extensive *ex vivo* expansion with high doses of IL-2 which may affect their stability, phenotype, and survival once transferred *in vivo*. It is thought that using IL-2 at low doses *in vivo* in combination with adoptive Treg cell therapy could promote Treg survival and improve therapeutic efficacy. Use of IL-2 in combination with adoptive Treg cell therapy has not been approved and clinical trials are underway investigating safety, dosing, and efficacy of low-dose IL-2 and Treg infusion for GvHD and T1D.^99–104^ More recently, it has also been clinically tested in stable liver transplant recipients patients 2–6 years post-transplant.^105^ Unfortunately, it is clear that using ld-IL-2 to increase Treg persistence is not without its risks, as there is clear evidence for activation of effector cells such as cytotoxic CD8^+^ T cells and NK cells in the recipient.^99,105^ In the context of SOTx, recently published results from a clinical trial (NCT02949492) that used ld-IL-2 to expand endogenous Tregs in LTx recipients reported rejection episodes in 4 of 5 participants who initiated immunosuppression withdrawal.^105^ These episodes were mild to moderate and tended to resolve after initiating immunosuppression. One participant, however, developed T cell mediated rejection that was unresponsive to steroids and the individual required re-transplantation.^105^ Due to these negative outcomes, it is very unlikely that ld-IL-2 can be easily developed as an adjuvant to Treg ACT supporting tolerance induction. As discussed below, however, several groups are developing IL-2 orthologs to target transferred Tregs more precisely without impact on other immune cells responsive to the natural endogenous cytokine.^106^

### Microparticles delivering chemokine CCL22

The rapid loss of infused Treg may also suggest that they undergo generalized and unfocused migration into the tissues or are lost in the absence of signals supporting their survival and required functions. As such, harnessing mechanisms that can orchestrate Treg ACT recruitment and function *in vivo* may be a promising new approach to improve clinical outcomes. In a recent proof-of-principle study, it was demonstrated that poly lactic-co-glycolic acid (PLGA) microparticles (MP) could generate a chemokine gradient of C-C-Motif Chemokine 22 (CCL22) to recruit CCR4-expressing Tregs *in vivo*.^107^ These CCL22 MP prolonged hindlimb allograft survival and promoted donor-specific tolerance.^108^ Additionally, synthetic human CCL22 MP induced human Treg migration *in vitro* demonstrating that this technology has the potential to improve Treg cell therapy efficacy by delivering chemokines, like CCL22, that directs them to the graft, or by providing other stimuli (i.e., IL-2, TGF-β) that support their survival or functions in the graft.

The use of IL-2, TGF-β, and rapamycin has been shown to favor Treg suppressive function and development.^109^ Currently, the use of low dose IL-2 in combination with Treg cell therapy is being evaluated to promote Treg persistence and endogenous Treg expansion.^99^ A limitation to this is that this approach is nonspecific and can have systemic off target effects. Additionally, a recently published study found that administration of low-dose IL-2 in liver Tx patients did increase the number of Tregs but failed to induce transplantation tolerance.^105^ An alternative to this would be to provide local extended release of these cytokines and drugs at the graft site to promote tolerance. In one study, Treg inducing microparticles (TRI-MP) were engineered to release TGF-β1, IL-2, and rapamycin to induce differentiation from naïve T cells.^110^ Using a rat hindlimb vascular composite allotransplantation (VCA) model, TRI-MP prolonged allograft survival without the use of immunosuppression. This system also enriched Treg and reduced inflammatory Th1 populations. The studies above suggest that microparticles can be engineered to support Treg localization, survival, and persistence after adoptive transfer to prevent allograft rejection and promote tolerance. Microparticles delivering supportive or stimulatory signals are an attractive potential method to add value to Treg ACT by boosting the efficacy of a product that, to date, has shown minimum impact.

### Engineering IL-2 cytokine-cytokine receptors

As described earlier, low dose IL-2 in combination with adoptive Treg transfer is an approach currently being evaluated in clinical trials to promote Treg survival and persistence. However, low dose IL-2 may support an inflammatory environment consisting of activated CD4^+^ and CD8^+^ cytotoxic effectors as well as NK cells making it difficult for Tregs to persist. Therefore, cytokine-cytokine receptor engineering such as the IL-2 cytokine receptor complex on Tregs may be an innovative approach to induce allograft tolerance and Treg survival without supporting effector T cell and NK cells that may contribute to GvHD response.

In a murine mixed chimerism model, FoxP3^+^GFP^+^-BALB/c Tregs (green fluorescent protein under the control of the mouse Foxp3 promotor) were transduced to express an orthogonal IL-2 (oIL-2) receptor β chain (oIL-2Rβ). Transduced Tregs were adoptively transferred along with C57BL/6 bone marrow cells (BMC) into wild type BALB/c recipients. Recipients that received oIL-2Rβ Tregs and treated with oIL-2 had significantly improved engraftment and increased percentage of FoxP3^+^GFP^+^ in CD4^+^ T cells without increasing CD8^+^ T cells. In addition, those same recipients had improved acceptance of heart allografts from C57BL/6 donor mice demonstrating donor-specific tolerance.^111^

Another study used a murine major mismatch acute GvHD model to investigate the suppressive function of this Treg orthogonal IL-2/IL-2 receptor complex. Briefly, irradiated BALB/c recipients received T cell-depleted BMC from C57Bl/6 mice with or without fresh C57Bl/6 FoxP3 Treg cells or with oIL-2Rβ Treg. On Day 2, C57Bl/6 T responder cells were injected to induce GvHD. Increased survival was observed in recipients that received oIL-2Rβ+ Tregs with oIL-2. Additionally, oIL-2 selectively expanded oIL-2Rβ Treg *in vivo* and retained their ability to migrate to the gastrointestinal tract and lymph nodes.^112^ These preclinical data support the potential of using cytokine-cytokine receptor engineering to improve adoptive Treg therapy efficacy for the treatment of Tx and GvHD. These data demonstrate the specificity of cytokine-cytokine receptor interactions avoiding interactions with the natural cytokine response, thus reducing potentially harmful off target expansion of effectors, improving allograft tolerance.

## Conclusions/future directions

**Figure 1.**
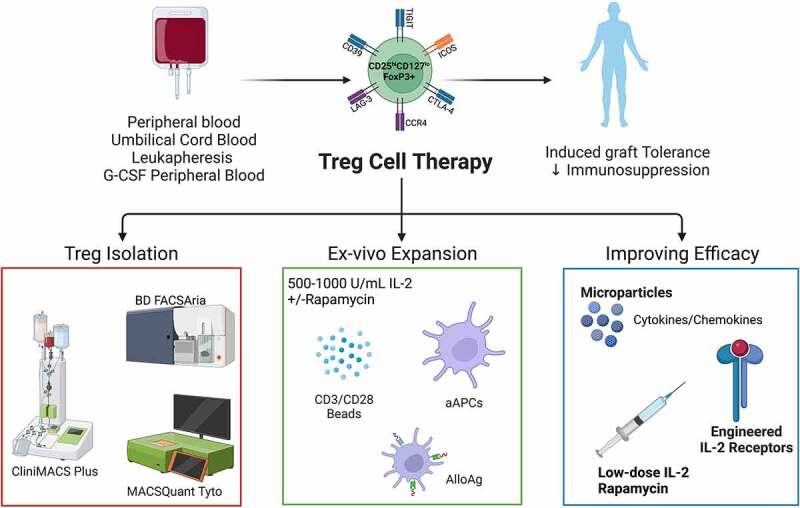
Overview of clinical manufacturing of regulatory T cell (Treg) products. Tregs can be isolated from peripheral blood, umbilical cord blood, leukapheresis, or G-CSF mobilized peripheral blood by magnetic cell separation and/or flow cytometric sorting using GMP grade closed systems. Isolated Tregs are then *ex vivo* expanded using anti-CD3/CD28 magnetic expander beads or artificial antigen-presenting cells (K562 64/86 aAPCs) in the presence of interleukin (IL)-2 with or without rapamycin. AlloAg-specific Tregs can be generated by culturing recipient Tregs with donor AlloAg-expressing APCs. Current *in vivo* and *ex vivo* strategies are being used to improve Treg ACT persistence and migration. Figure created with BioRender.com.

Overall, the steps for human Treg isolation and *ex vivo* expansion for clinical use have been outlined here and in other reviews.^24,44,52,113^ However, it is clear from this discussion that there is still work required to get to the generation of a functionally therapeutic product for clinical use that matches the effectiveness observed in preclinical studies. Clinical trials using Treg-based therapeutics for the prevention of GvHD after HCT or rejection after SOTx have been described and consistently shown that infusion of *ex vivo* expanded Tregs is well tolerated and safe.^24,44,52^ Technical advancements in clinical manufacturing of Treg cell products for ACT have improved the isolation of these cells, with many coming online that allow isolation in closed systems as part of GMP processes. This includes positive selection for CD25 followed by subsequent sorting for CD4^+^CD127^lo^CD25^hi^ using Miltenyi’s closed system magnetic cell separator (CliniMACS Plus) and sorter (MACSQuant Tyto) (Figure 1). Variations in the approach and reagents used for *ex vivo* expansion indicate that optimization and standardization are still needed to enable consistent generation of functionally stable Tregs for clinical use, and are summarized in Figure 1. Additionally, variation in manufacturing and reporting characteristics of Treg cell products may be a barrier to effectively compare study results between institutions and hamper future product development. Therefore, efforts have been made in this review to outline the minimum information required to interpret and compare experimental findings.^113^ This may help standardize future studies allowing for more effective comparison and reproducibility. Lastly, results from clinical trials have driven novel preclinical studies aiming to improve the clinical application of Tregs. Studies include developing modified cytokine-cytokine receptor complexes to promote *in vivo* persistence and migration to target graft sites or lymphatic tissues (see Figure 1).
